# CD147 expression predicts biochemical recurrence after prostatectomy independent of histologic and pathologic features

**DOI:** 10.1186/s12885-015-1559-4

**Published:** 2015-07-25

**Authors:** Tyler M. Bauman, Jonathan A. Ewald, Wei Huang, William A. Ricke

**Affiliations:** 1 Departments of Urology ,Carbone Cancer Center, University of Wisconsin, 7107 Wisconsin Institutes of Medical Research (WIMR), 1111 Highland Ave., 53705 Madison, WI USA; 2 Departments of Pathology and Laboratory Medicine, University of Wisconsin, Madison, WI USA; 3 University of Wisconsin O’Brien Urology Research Center, University of Wisconsin, Madison, WI USA; 4 Carbone Cancer Center, University of Wisconsin, Madison, WI USA

**Keywords:** Antigen, CD147, Recurrence, Prostatectomy, Biological marker

## Abstract

**Background:**

CD147 is an MMP-inducing protein often implicated in cancer progression. The purpose of this study was to investigate the expression of CD147 in prostate cancer (PCa) progression and the prognostic ability of CD147 in predicting biochemical recurrence after prostatectomy.

**Methods:**

Plasma membrane-localized CD147 protein expression was quantified in patient samples using immunohistochemistry and multispectral imaging, and expression was compared to clinico-pathological features (pathologic stage, Gleason score, tumor volume, preoperative PSA, lymph node status, surgical margins, biochemical recurrence status). CD147 specificity and expression were confirmed with immunoblotting of prostate cell lines, and CD147 mRNA expression was evaluated in public expression microarray datasets of patient prostate tumors.

**Results:**

Expression of CD147 protein was significantly decreased in localized tumors (pT2; *p* = 0.02) and aggressive PCa (≥pT3; *p* = 0.004), and metastases (*p* = 0.001) compared to benign prostatic tissue. Decreased CD147 was associated with advanced pathologic stage (*p* = 0.009) and high Gleason score (*p* = 0.02), and low CD147 expression predicted biochemical recurrence (HR 0.55; 95 % CI 0.31–0.97; *p* = 0.04) independent of clinico-pathologic features. Immunoblot bands were detected at 44 kDa and 66 kDa, representing non-glycosylated and glycosylated forms of CD147 protein, and CD147 expression was lower in tumorigenic T10 cells than non-tumorigenic BPH-1 cells (*p* = 0.02). Decreased CD147 mRNA expression was associated with increased Gleason score and pathologic stage in patient tumors but is not associated with recurrence status.

**Conclusions:**

Membrane-associated CD147 expression is significantly decreased in PCa compared to non-malignant prostate tissue and is associated with tumor progression, and low CD147 expression predicts biochemical recurrence after prostatectomy independent of pathologic stage, Gleason score, lymph node status, surgical margins, and tumor volume in multivariable analysis.

**Electronic supplementary material:**

The online version of this article (doi:10.1186/s12885-015-1559-4) contains supplementary material, which is available to authorized users.

## Background

An estimated 238,590 new cases of prostate cancer (PCa) are expected in 2013, accounting for 28 % of total malignancies in men [1]. After surgery, some patients experience biochemically recurrent disease, resulting in unfavorable prognosis and an estimated cancer-specific mortality of 11–22 % [2]. Treatment options are limited for patients that progress to metastatic disease, as androgen ablation inevitably leads to lethal castration-resistant prostate cancer. The identification of prognostic factors that predict metastatic recurrence after prostatectomy would allow the stratification of at-risk patients and assist in more effective individualized treatment. The reliability of the currently accepted biomarker used to detect PCa recurrence, prostate-specific antigen (PSA), has recently been questioned [3], and highlights the urgent need for more effective PCa-specific biomarkers to guide patient treatment.

Matrix metalloproteinases (MMPs) are proteinases that play a role in tissue remodeling and disease progression through regulation of the extracellular microenvironment. Extracellular matrix metalloproteinase inducer CD147 (EMMPRIN, basigin) is a membrane-associated glycoprotein that stimulates the synthesis of several MMPs, including interstitial collagenase (MMP-1), gelatinase A (MMP-2), stromelysin-1 (MMP-3), and gelatinase B (MMP-9) [4–6]. Elevated CD147 expression has been previously associated with cancer progression and invasion in malignancies such as breast and colorectal cancer [7, 8].

Previous studies have observed similar trends in PCa, with increased CD147 expression in PCa samples compared to matched benign samples and an association with poor prognosis after prostatectomy [9–13]. However, two recent studies have reported contrary results, with a decrease in CD147 observed with PCa progression [13, 14], suggesting that CD147 may play a protective role in PCa. The prognostic role of CD147 in PCa is still debated, as results thus far are mixed [11–14]. Furthermore, the sensitivity of previous studies has been limited due to semi-quantitative methods of analyzing immunohistochemical staining. The purpose of this study was to investigate the prognostic role of CD147 in PCa and the expression of CD147 in PCa progression using quantitative multispectral imaging.

## Methods

### Patient cohort and tissue microarray

The University of Wisconsin Institutional Review Board (IRB) (M-2007-110-CP003) approved retrospective review of patient information and demographics and provided ethical insight to this study in accordance with local and university policies, state laws, and federal regulations. Patient consent was not deemed necessary because issues were obtained from a pathology archive and patient identifying information was anonymized and de-identified prior to analysis.

The PCa progression tissue microarray (pTMA) and outcomes tissue microarray (oTMA) were constructed as previously described [15, 16]. Briefly, all samples were collected at the University of Wisconsin Hospital from 1985–2005. University of Wisconsin pathologists issued pathological reports at the time of surgery, and an expert genitourinary pathologist (WH) extracted pathological characteristics from the reports retrospectively when constructing the TMAs. The pTMA includes 384 duplicate cores representing tumor-adjacent normal prostate (BPT; *n* = 96 cores), benign prostatic hyperplasia (BPH; *n* = 48), high-grade prostatic intraepithelial neoplasia (HGPIN; *n* = 50), localized PCa (*n* = 86), aggressive PCa (*n* = 60), and metastases (*n* = 44). The oTMA includes 462 duplicate cores from normal prostate (*n* = 96), non-recurrent PCa (*n* = 250), and recurrent PCa (*n* = 116). Diagnosis for each core was confirmed by a genitourinary pathologist (WH). Tumor volume values were obtained from radical prostatectomy reports. Positive margins were defined as tumor invasion <1mm from the inked margin. All outcomes patients had ≥5 years of regular follow-up and no signs of metastatic disease at time of surgery. Patients included in outcomes analysis had PSA levels nadir to undetectable levels after prostatectomy, and recurrence was defined in this analysis as PSA biochemical recurrence above 0.2 ng/ml or local/metastatic recurrence within follow-up period, as has been previously published [15, 16].

### Immunohistochemistry

Samples were processed and stained by immunohistochemistry (IHC) as previously described [16, 17]. Tissues were stained using mouse monoclonal anti-CD147 (Meridian Life Science, Memphis, TN; 1:75 in Renoir Red [Biocare, Concord, CA]), mouse monoclonal anti-E-caderin (Dako, Carpinteria, CA; 1:200 in Biocare Renoir Red), and Mach 2 Mouse HRP-Polymer (Biocare) as a secondary antibody. Bajoran Purple chromogen (Biocare) was used to detect CD147 and Deep Space Black (Biocare) was used to detect E-caderin.

### Image analysis

Data acquisition and image analysis was performed using the Vectra slide scanner (PerkinElmer, Waltham, MA), Nuance software (PerkinElmer) and inForm software (PerkinElmer), as previously described [16, 17]. CD147 expression and E-cadherin expression were used to identify and segment epithelial cell plasma membrane, and hematoxylin was used to segment nuclei (Additional file 1: Figure S1). CD147 and E-cadherin expression were then quantified in the membrane and cytoplasm of the epithelium. Cores with significant folding or <5 % epithelium were eliminated from analysis. The average mean OD of duplicate cores was used for analysis if both cores were available and sufficient for analysis.

### Statistical analysis

Differences in CD147 protein expression were assessed using the Student’s t-test or one-way ANOVA with multiplicity adjusted p-values (normal distribution). Mann-Whitney or Kruskal-Wallis tests were performed for non-Gaussian distributions. Cox proportional hazards regression was used to investigate the prognostic ability of CD147 and E-cadherin, along with clinical and pathologic variables, including age at surgery, initial pre-surgical serum PSA, Gleason score, tumor volume, pathologic stage, lymph node status, and surgical margins. Kaplan-Meier curves for membranous CD147 and E-cadherin expression were constructed based on separation at the median of expression for PCa patients only, and the log-rank test was used to compare outcomes in Kaplan-Meier analysis. A multivariable model was constructed using biomarker expression and clinico-pathological variables, and the assumption of proportional hazards was checked using Kolmogorov-Smirnov type supremum tests. Because expression of CD147 was not normally distributed, the median of CD147 expression was used to divide patients in multivariable analysis. Using logistic regression, a clinico-pathological model for predicting 5-year biochemical recurrence-free survival was created and the area under the curve (AUC) was calculated. CD147 was then incorporated into this model and a new AUC was calculated. MedCalc v11.4 (Ostend, Belgium) was used for statistical analysis and a two-sided p-value <0.05 was considered significant in all analyses.

### Publicly available microarray data

Expression of CD147-coding gene *BSG* mRNA was assessed using public expression microarray dataset GSE21034 [18] and GSE25136 [19], available from National Center for Biotechnology Information Gene Expression Omnibus [20]. *BSG* expression data was collected from each dataset, averaged and compared based on Gleason score, pathologic stage, and recurrence status using previously described methods [21].

### Immunoblot analysis of PCa progression model cells

In order to investigate the specificity of the CD147 antibody, we used immunoblot analysis of prostate cell lines. The human non-tumorigenic prostate epithelial cell line BPH-1 and a xenograft-derived BPH1 tumor cell line (T10) were cultured as previously described [22, 23]. Cells were solubilized and proteins were analyzed by immunoblot analysis using mouse anti-CD147 (Meridian Life Science, Memphis, TN), and anti-Actin (Santa Cruz Biotechnology, Dallas, TX) with anti-mouse HRP-conjugated secondary antibodies, as previously described [24].

## Results

### Cellular localization of CD147 expression

**Fig. 1:**
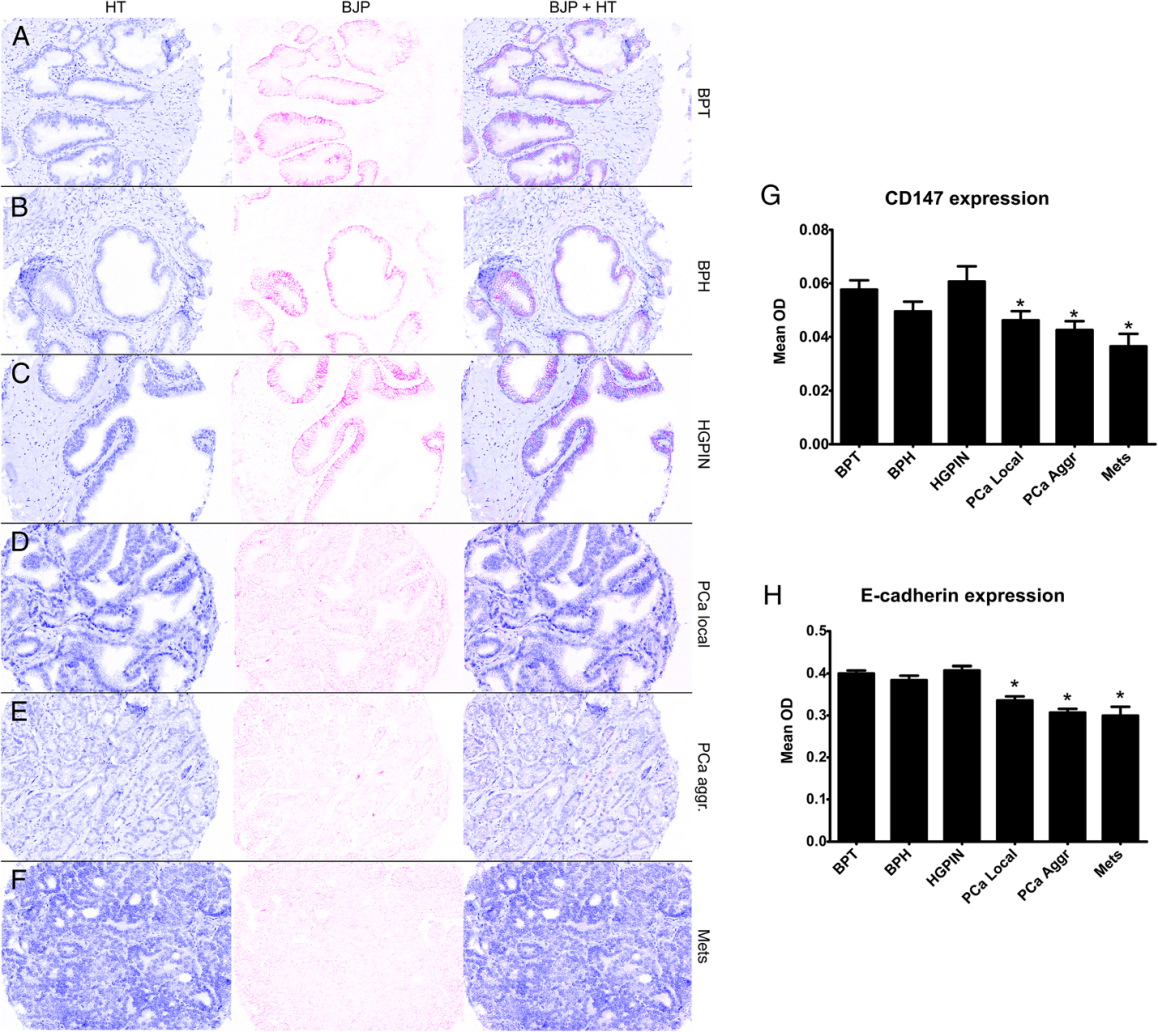
Bajoran Purple (BJP) chromogen was used to mark CD147 expression in prostate samples. BJP was separated from the hematoxylin (HT) counterstain using inForm software (**a-f**) and was then quantified in the plasma membrane (**g**). No significant differences were observed between benign prostatic tissue (BPT; *n* = 46 patients) and benign prostatic hyperplasia (BPH; *n* = 23) samples (*p* = 0.15) or high-grade intraepithelial neoplasia (HGPIN; *n* = 24; *p* = 0.63). Significant decreases in expression were observed in localized prostate cancer (PCa local; *n* = 42; *p* = 0.02), aggressive prostate cancer (PCa aggr.; *n* = 31; *p* = 0.004), and metastases (Mets; *n* = 20; *p* = 0.001) compared to BPT. E-cadherin was quantified for validation of membrane segmentation (**h**), and significant decreases in expression were found in all PCa samples (p < 0.0001) compared to BPT but not in BPH or HGPIN (p > 0.05) *p < 0.05

In preliminary studies, individual sections of a limited sample of patient PCa tumor tissues were stained for CD147 and by IHC to optimize staining, visualization, and quantification of CD147 and E-cadherin. CD147 expression was primarily detected in the plasma membrane (Fig. 1 and Additional file 1: Figure S1). The measured expression of membrane-associated CD147 (average mean OD ± SD: 0.027 ± 0.011) was 3.3-fold higher than in the cytoplasm (0.008 ± 0.006; p <0.0001). Therefore, membrane-associated CD147 was measured and reported. In total, 41 of 384 (10.6 %) of cores in the pTMA were removed from analysis due to folding or <5 % epithelium, and 18 of 462 (3.9 %) cores were removed from the oTMA.

### Expression of CD147 and E-cadherin in PCa progression

CD147 expression in BPT, BPH, HGPIN, and PCa tumors was quantified, and all groups were compared to BPT (Fig. 1). Membrane-associated E-cadherin expression was significantly decreased in localized PCa (p < 0.0001), aggressive PCa (p < 0.0001), and metastases (p < 0.0001). No difference was observed between benign prostate tissue and BPH or HGPIN samples. Membrane-associated CD147 was significantly decreased in localized PCa (*p* = 0.02), aggressive PCa (*p* = 0.004), and metastases (*p* = 0.001). No significant change was observed in BPH or HGPIN. These results suggest that CD147 expression is decreased PCa, with lower expression in advanced tumors and metastases.

### Relationship of CD147 expression to tumor pathology

To determine whether decreased CD147 expression was associated with clinico-pathologic characteristics and patient outcomes (Table 1), we assessed prostate cancer specimens using quantitative multispectral imaging. CD147 expression was evaluated in relation to tumor stage. We found that CD147 expression was significantly decreased in T3b tumors compared to pT2 tumors (*p* = 0.009), but pT3a tumors were not significantly different than pT2 (*p* = 0.77). Decreased CD147 expression also associated with high Gleason scores (*p* = 0.02). Additionally, patients experiencing biochemical recurrence within the follow-up period had significantly lower expression of CD147 than non-recurrent PCa patients (*p* = 0.001). There was no significant relationship between CD147 and tumor volume, presence of positive surgical margins, or lymph node status. These results suggest that decreased CD147 expression is associated with some pathologic features of advanced PCa.

**Table 1:** Membrane-associated CD147 expression and patient demographics for PCa outcomes analysis

	Number (%)	Mean OD (±SD)	p-value
Pathologic stage			
pT2	143 (78.1)	0.027 (±0.010)	-
pT3a	21 (11.5)	0.027 (±0.009)	0.77
pT3b	19 (10.4)	0.021 (±0.007)	0.009
Gleason score			
≤6, 3 + 4	143 (78.1)	0.028 (±0.010)	-
4 + 3, ≥8	40 (21.9)	0.023 (±0.009)	0.02
Preoperative PSA (ng/ml)			
<4	24 (13.1)	0.026 (±0.011)	-
4–10	125 (68.3)	0.027 (±0.010)	0.84
>10	34 (18.6)	0.025 (±0.008)	0.87
Tumor volume			
<10 %	40 (22.1)	0.026 (±0.011)	-
10–19 %	42 (23.2)	0.029 (±0.011)	0.82
20–29 %	29 (16.0)	0.029 (±0.010)	0.27
30–39 %	33 (18.2)	0.026 (±0.009)	0.77
≥40 %	37 (20.4)	0.024 (±0.010)	0.45
Recurrence status			
Non-recur	125 (68.3)	0.028 (±0.011)	-
Recur	58 (31.7)	0.023 (±0.008)	0.001
Positive margins			
No	88 (48.9)	0.027 (±0.010)	-
Yes	92 (51.1)	0.026 (±0.010)	0.53
Lymph node status			
Negative	169 (95.5)	0.027 (±0.010)	-
Positive	8 (4.5)	0.022 (±0.009)	0.15

Abbreviations: prostate cancer (PCa), prostate-specific antigen (PSA), optical density (OD)

### CD147 and post-surgical prognosis

**Fig. 2:**
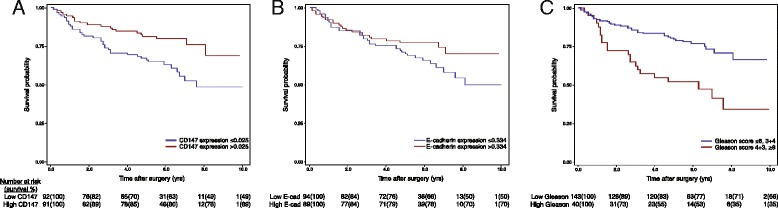
Kaplan-Meier estimates of disease recurrence by separation of patients at the median of membrane-associated CD147 expression (**a**; p < 0.05), membrane-associated E-cadherin expression (**b**), and Gleason score (**c**; p < 0.05)

The ability of CD147 and clinico-pathologic characteristics to predict biochemical recurrence after prostatectomy was assessed in 183 patients on the outcomes TMA (Table 2). Median follow-up for all patients was 6.2 years (IQR 5.5-7.4). Lower E-cadherin expression resulted in poor prognosis after prostatectomy (HR 0.41; CI 0.24–0.68; *p* = 0.0007). Patients with lower CD147 expression performed significantly worse (0.61; 0.44–0.82; *p* = 0.002). Gleason score (*p* = 0.005), tumor volume (*p* = 0.02), pathologic stage (p < 0.0001), and lymph node status (*p* = 0.007) were significant predictors in univariable analysis. Age at surgery (*p* = 0.78), surgical margins (*p* = 0.07), and initial serum PSA (*p* = 0.90) were not predictive of recurrence. Membrane-associated CD147 and Gleason score were predictive of biochemical recurrence in Kaplan-Meier log-rank analysis (Fig. 2). In bivariable analysis, CD147 expression was associated with biochemical recurrence (0.51; 0.29–0.91; *p* = 0.02) independent of E-cadherin expression (*p* = 0.46; data not shown). Lower CD147 expression was predictive of biochemical recurrence (0.55; 0.31–0.97; *p* = 0.04) independent of pathologic stage, Gleason score, lymph node status, and surgical margins in multivariable analysis (Table 3), and no variables included in the model violated the proportional hazards assumption (p > 0.05). The AUC for predicting 5-year biochemical recurrence-free survival in a model incorporating lymph node status, Gleason score, and pathologic stage was 0.73. When CD147 staining was incorporated, the AUC increased to 0.76.

**Table 2:** Univariable analysis of ability to predict disease recurrence

	Hazard Ratio	95 % CI for HR	p-value
Age	1.01	0.97–1.04	0.78
Gleason score			0.005
≤6, 3 + 4	ref		
4 + 3, ≥8	2.59	1.53–4.40	
Tumor volume			0.02
<10 %	ref		
10–19 %	1.49	0.57–3.90	
20–29 %	1.42	0.50–4.03	
30–39 %	2.88	1.18–7.05	
≥40 %	3.30	1.39–7.84	
Pathologic stage			<0.0001
T2	ref		
T3a	3.61	1.85–7.05	
T3b	6.73	3.68–12.29	
Positive lymph node(s)	3.58	1.62–7.89	0.007
Initial PSA (per ng/ml)	1.00	0.99–1.01	0.90
Positive surgical margins	1.63	0.96–2.77	0.07
CD147 expression (per 0.01 OD)	0.61	0.44–0.82	0.002
E-cadherin expression (per 0.1 OD)	0.41	0.24–0.68	0.0007

Abbreviations: prostate-specific antigen (PSA), reference (ref), optical density (OD)

**Table 3:** Multivariable analysis of ability to predict disease recurrence

	Hazard Ratio	95 % CI for HR	p-value
Gleason score			
≤6, 3 + 4	ref		
4 + 3, ≥8	1.57	0.89–2.76	0.12
Pathologic stage			
T2	ref		
T3a	3.46	1.73–6.92	0.0005
T3b	4.12	2.06–8.26	0.0001
Positive lymph node(s)	1.48	0.65–3.40	0.36
Positive surgical margins	1.25	0.70–2.22	0.46
CD147 expression			
≤0.025	ref		
>0.025	0.55	0.31–0.97	0.04

Abbreviations: reference (ref)

### CD147 protein in human prostate cell lines

**Fig. 3:**
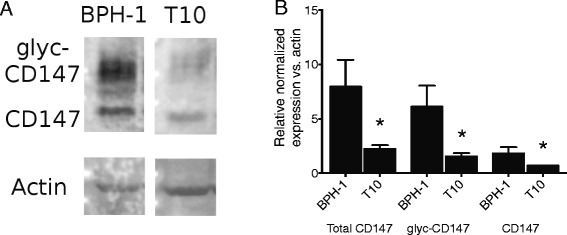
Normalized protein lysates of non-tumorigenic BPH-1 cells and xenograft tumor-derived T10 cells were analysed by Western immunoblot (**a**). Actin expression is shown as a control, and error bars represent the standard deviation from three independent experiments. Total CD147 expression was significantly decreased in tumor-derived T1 (*p* = 0.02) cells compared to non-tumorigenic BPH-1 cells. Similarly, glycosylated CD147 was significantly reduced in T10 cells (*p* = 0.02)

Next, CD147 protein was evaluated in the non-tumorigenic prostate epithelial cell line BPH-1 and human xenograft-derived T10 primary metastatic PCa tumor cell line. Bands were detected at 44 kDa and 66 kDa in both cell lines, representing non-glycosylated and glycosylated forms of CD147 (Fig. 3a). These results indicate that our antibody is specific for known isoforms of CD147. After normalizing to actin, total CD147 expression was significantly decreased in tumor-derived T1 (*p* = 0.02) cells compared to non-tumorigenic BPH-1 cells (Fig. 3b). Furthermore, both glycosylated and non-glycoslated CD147 were decreased in T10 cells compared to BPH-1 cells (*p* = 0.02 for both). These data suggest an inverse relationship between CD147 expression and tumorigenicity in a T + E2-transformed BPH-1 xenograft model.

### BSG expression in publicly available microarrays

Expression microarray data from previously published studies were used to address whether PCa stage- or recurrence-associated decreases in CD147-encoding *BSG* expression are transcriptionally regulated. These data were originally generated to investigate gene expression changes in patient prostate tumors in respect to Gleason Score and tumor stage [18] and PCa recurrence [19]. In analysis of PCa samples, decreased *BSG* expression was associated with Gleason score ≥8 (*p* = 0.008) and pathologic stage pT3b (*p* = 0.03), but not with post-surgical recurrence (*p* = 0.26; Table 4). These results suggest that decreases in CD147 protein expression in advanced PCa may be transcriptionally regulated.

**Table 4:** Evaluation of CD147-encoding *BSG* expression in PCa using publically available microarray data

Characteristic	Dataset	Number	Mean (±SD)	p-value
Pathologic stage	GSE21034			
pT2		86	10.17 (±0.34)	-
pT3a		30	10.18 (±0.28)	0.88
pT3b,c		17	9.97 (±0.40)	0.03
Gleason score	GSE21034			
6		77	10.21 (±0.34)	-
7		42	10.13 (±0.29)	0.22
≥8		11	9.91 (±0.34)	0.008
Recurrence status	GSE21356			
Non-recur		40	7.81 (±0.54)	-
Recur		39	7.68 (±0.52)	0.26

Abbreviations: prostate cancer (PCa)

## Discussion

CD147 is a multifunctional glycoprotein that acts to induce the expression of MMPs, among other roles [4–6, 25]. CD147 also forms heterodimers with proton-coupled monocarboxylate transporters (MCT) MCT1, MCT3, and MCT4 [13, 25], helping to maintain lactate and pH homeostasis in epithelial cells, while N-glycosylation of CD147 leads to self-aggregation and MMP induction [26]. Overexpression of CD147 of MMPs and CD147 has been noted in many malignancies, including breast and colorectal cancer [7, 8], as the mechanistic role of highly glycosylated CD147 is suspected to assist in tumor invasion and metastatic spread [26, 27]. However, the prognostic role of CD147 is still controversial, particularly in PCa.

Our results show that CD147 is decreased in malignant prostate samples compared to tumor-adjacent normal tissue, and further decreases in expression are associated with advanced pathologic stage and Gleason score, indicating that CD147 may be important in the progression to advanced stages of PCa. Using a xenograft-derived cell line model of prostate cancer progression, we confirmed that CD147 expression was decreased in aggressive PCa. Investigation of CD147-encoding *BSG* mRNA expression in publicly available microarray data shows that CD147 protein changes may be regulated transcriptionally. In this study, we also found that CD147 predicts biochemical recurrence after prostatectomy.

Our results are in disagreement with earlier studies on the expression and prognostic role of CD147 in PCa [9–13]. However, one recent study of 11,152 patients found a decrease in CD147 expression between PCa and BPT and with increasing stage and Gleason score [14], and these results are in agreement with one earlier study on CD147 expression in PCa [13]. However, these studies concluded that CD147 does not play a significant prognostic role in determining post-surgical PSA recurrence [14] or that PCa patients with higher CD147 expression performed significantly worse [13]. This is the first study, to our knowledge, to demonstrate that low expression of CD147 is indicative of poor prognosis after prostatectomy independent of clinico-pathological features.

One limitation of previous studies is the semi-quantitative approach of evaluating immunohistochemical staining. While these methods are generally effective in quantitating “on/off” proteins, this approach is less effective when analyzing proteins with a heterogeneous staining pattern, particularly when proteins are localized to the membrane or cytoplasm, as small differences are not readily detected using manual methods of quantification. In this study, we show that CD147 is primarily localized to the cellular membrane. Quantitation of E-cadherin in the membrane portion of the epithelium resulted in a significant decrease in expression in all PCa samples compared to benign prostatic tissue, while HGPIN and BPH samples showed no significant differences in expression. Furthermore, membrane-associated E-cadherin expression predicted biochemical recurrence after prostatectomy in our patient cohort. This data is in agreement with previous studies on E-cadherin in PCa [16, 28], and thus validates the epithelial cell membrane segmentation for investigation of CD147.

Antibodies to N-terminal synthetic peptides or recombinant fragments of CD147 have shown predominant localization to the basal and lateral plasma membrane, and studies using these antibodies have shown similar decreases in CD147 expression along PCa progression [13, 14]. As has been argued previously [14], the use of different antibodies may account for some of the contrasting results found in current literature on CD147. Some previous studies have shown cytoplasmic and nuclear staining of CD147 in PCa specimens [11], rather than expected membrane-associated localization. Like previous studies with similar localization and expression results [13, 14], CD147 protein expression in our cohort of patient samples was largely present in the plasma membrane. Additionally, our immunoblot results showed bands at molecular weights consistent with both glycosylated and nonglycosylated forms of CD147. When considering the known roles and expected localization of CD147 [5, 27], our results suggest high antibody specificity for known forms of CD147.

Our results differ from previous studies on CD147 and other malignancies [7, 8]. This may be attributable to the diverse roles and unusually high amount of post-translational modifications to CD147. The mechanistic role of CD147 of inducing MMP expression and promoting extracellular matrix degradation and reconstruction has implicated CD147 in tumor invasion. Caveolin-1 is a putative tumor suppressor in other malignancies [26, 29, 30] that binds CD147 and suppresses N-glycosylation and CD147-induced fibroblast MMP activation. Caveolin-1 serves a unique role in PCa, as secretion and overexpression of caveolin-1 is associated with advanced disease [31]. Though the interaction of caveolin-1 and CD147 was not investigated in this study, the overexpression of caveolin-1 in PCa progression may serve to bind CD147 and preventing N-glycosylation, self-aggregation, and MMP induction, thus accounting for the decrease in CD147 expression in advanced PCa that we observed.

Furthermore, the N-terminal domain of CD147 is essential for MMP induction [27], as N-glycosylation of CD147 is associated with self-aggregation and MMP expression [26]. In HT1080 fibrosarcoma and A431 epidermoid carcinoma cells, purification of a 22-kDa CD147 fragment from culture medium demonstrated cleavage and shedding of CD147 by membrane-type MMPs in the linking region between the two Ig-like domains [27]. This soluble 22-kDa fragment was adequate for augmentation of MMP-2 expression in human fibroblasts, while the cleavage of membrane CD147 is expected to downregulate the membrane-specific MMP-activating function of CD147 [27]. N-terminal cleavage and shedding of CD147 has not yet been studied in PCa, but may provide a mechanistic explanation for the decrease in CD147 with PCa progression that was observed in this and previous studies [13, 14], as antibodies to the N-terminal Ig1 domain may not recognize cleaved forms of CD147. In this study, we also found that *BSG* mRNA expression was not significantly associated with recurrence, indicating that post-translational modifications of CD147 may be important in the metastatic spread of PCa. Further studies are needed on post-translational modified forms of CD147 in PCa to reconcile the opposing results found throughout the literature.

Limitations of this study include both sample size and the use of tumor-adjacent normal as a baseline for comparison of expression. Compared to previous studies [14] our sample size is relatively low, but the use of a quantitative multispectral platform increases the sensitivity of our assay and helps to make our sample size sufficient to draw conclusions. The use of tumor-adjacent normal tissues is common practice due to the difficult of obtaining truly normal tissues. However, future studies should incorporate autopsy normal prostate tissues to avoid the well-documented field effects of tumors.

## Conclusions

Membrane-associated CD147 expression significantly decreases between benign and malignant prostate samples but not BPH, and decreases in expression are further associated with increasing Gleason score and pathologic stage, suggesting an association between advanced PCa and decreased CD147 expression. CD147 staining is predictive of biochemical recurrence after prostatectomy independent of pathologic stage, surgical margins, Gleason score, lymph node status, and tumor volume.

## Additional file

Additional file 1: Figure S1. Using inForm software (PerkinElmer), 18 % of original images (A) were used to create an algorithm of differentiation to segmental epithelial and stromal components (B). E-cadherin was used to assist in cellular segmentation (C) of the membrane portion (D). Expression of CD147 was largely localized to the plasma membrane (E), as indicated by arrows. (TIFF 18051 kb)

## Abbreviations

(PCa): Prostate cancer
(PSA): Prostate-specific antigen
(MMP): Matrix metalloproteinase
(TMA): Tissue microarray
(IHC): Immunohistochemistry
(BPT): Benign prostatic tissue
(BPH): Benign prostatic hyperplasia
(HGPIN): High-grade prostatic intraepithelial neoplasia
(MCT): monocarboxylate transporter
