# Supplementary figures and images for: CD147 expression predicts biochemical recurrence after prostatectomy independent of histologic and pathologic features
Source: BMC Cancer. 2015 Jul 25;15:549. doi: 10.1186/s12885-015-1559-4 (PMC4514016; doi:10.1186/s12885-015-1559-4)

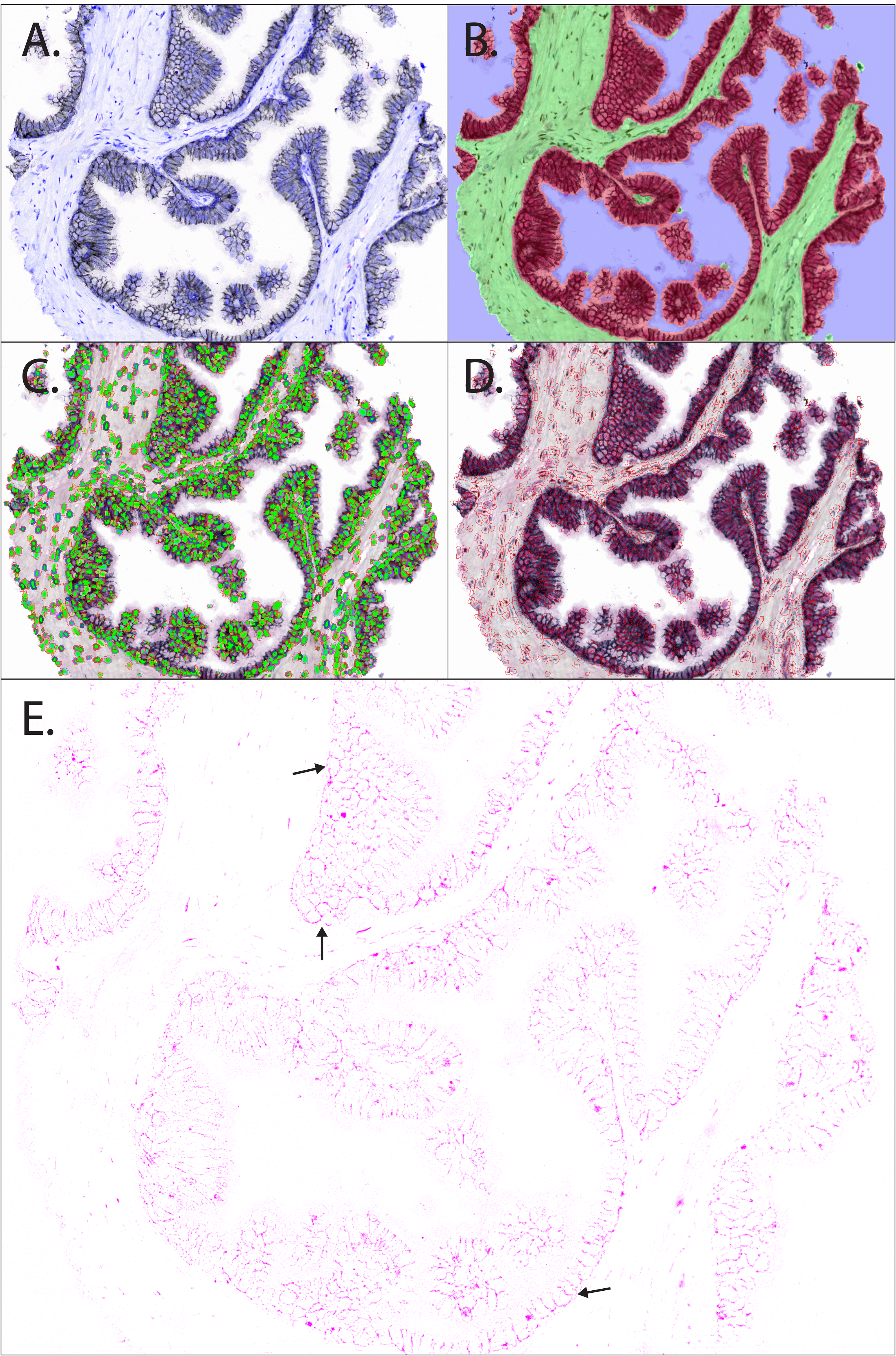

Supplement: Additional file 1: Figure S1. — Using inForm software (PerkinElmer), 18 % of original images (A) were used to create an algorithm of differentiation to segmental epithelial and stromal components (B). E-cadherin was used to assist in cellular segmentation (C) of the membrane portion (D). Expression of CD147 was largely localized to the plasma membrane (E), as indicated by arrows. (TIFF 18051 kb) [file 12885_2015_1559_MOESM1_ESM.tiff]
